# Automated quantification of anterior chamber cells using swept-source anterior segment optical coherence tomography

**DOI:** 10.1186/s12348-025-00456-y

**Published:** 2025-01-09

**Authors:** Shani Pillar, Shin Kadomoto, Keren Chen, Saitiel Sandoval Gonzalez, Nina Cherian, Joseph K. Privratsky, Nicolette Zargari, Nicholas J. Jackson, Giulia Corradetti, Judy L. Chen, SriniVas R. Sadda, Gary N. Holland, Edmund Tsui

**Affiliations:** 1 Ocular Inflammatory Disease Center, UCLA Jules Stein Eye Institute, Los Angeles, USA; 2 Department of Ophthalmology, David Geffen School of Medicine at UCLA, University of California, Los Angeles, Los Angeles, CA USA; 3 Department of Ophthalmology and Visual Sciences, Kyoto University Graduate School of Medicine, Kyoto, Japan; 4 Department of Medicine, Statistics Core, David Geffen School of Medicine at UCLA, University of California, Los Angeles, Los Angeles, CA USA; 5 Doheny Eye Institute, Pasadena, CA USA; 6 UCLA Jules Stein Eye Institute, 100 Stein Plaza, Los Angeles, CA 90095-7000 USA

**Keywords:** Uveitis, Anterior chamber inflammation, Optical coherence tomography (OCT), Image analysis, Standardization of Uveitis nomenclature (SUN)

## Abstract

**Purpose:**

To validate automated counts of presumed anterior chamber (AC) cells in eyes with histories of uveitis involving the anterior segment using swept-source (SS) anterior segment optical coherence tomography (AS-OCT) against manual counts and compare automated counts against Standardized Uveitis Nomenclature (SUN) criteria.

**Methods:**

Eyes were imaged with the ANTERION SS AS-OCT device (Heidelberg Engineering). A fully automated custom algorithm quantified the number of hyper-reflective foci (HRF) in line-scan images. Automated and manual counts were compared using interclass correlation (ICC) and Pearson correlation coefficient. Automated counts were compared to SUN grades using a mixed-effects linear regression model.

**Results:**

90 eyes (54 participants) were included; 67 eyes (41 participants) had histories of uveitis, while 23 eyes (13 healthy participants) served as controls. ICC comparing automated to manual counts was 0.99 and the Pearson correlation coefficient was 0.98. Eyes at each SUN grade with corresponding median HRF (interquartile range [IQR]) were: Grade 0, 42 eyes, 2 HRF (0,4); 0.5+, 10 eyes, 10 HRF (8,15); 1+, 9 eyes, 22 HRF (15,33); 2+, 3 eyes, 27 HRF; 3+, 2 eyes, 128 HRF; 4+, 1 eye, 474 HRF. For every 1-step increase in grade, automated count increased by 38 (*p* < 0.001) or 293% (Pearson correlation coefficient 0.80, *p* < 0.001). Automated counts differed significantly between clinically inactive eyes (2 HRF [0,4]) and controls (0 HRF [0,1], *p* = 0.02).

**Conclusions:**

Our algorithm accurately counts HRF when compared to manual counts, with strong correlation to SUN clinical grades. SS AS-OCT offers the advantage of imaging of the entire AC and may allow detection of subclinical inflammation in eyes that appear clinically inactive.

**Supplementary Information:**

The online version contains supplementary material available at 10.1186/s12348-025-00456-y.

Anterior chamber (AC) cells are a hallmark of inflammation that involves the anterior segment of the eye [1]. Detection and quantification of AC cells is used to evaluate disease severity and to monitor response to anti-inflammatory therapy. Accurate quantification of cells is therefore critical for the management of patients with uveitis [2]. Slit-lamp biomicroscopy, using the Standardized Uveitis Nomenclature (SUN) grading system is the current method by which most clinicians quantify AC cells [3], but this technique has substantial limitations. Biomicroscopy is observer- and instrument-dependent, and the ordinal SUN grading system may not be sufficiently sensitive to detect small, but clinically important, changes in levels of inflammation [4, 5]. Lack of a linear measure for AC cells could mean that within-grade changes will be overlooked during clinical assessment.

Instrument-based techniques using anterior segment optical coherence tomography (AS-OCT) have shown potential for providing an objective assessment of AC cells, with good correlation to clinician-assigned SUN grades [6, 7]. The ANTERION device (Heidelberg Engineering, Heidelberg, Germany) is a high-resolution swept-source (SS) AS-OCT. When compared to spectral domain AS-OCT devices, its longer wavelength and its improved scanning speed and scanning density result in a high-resolution image of the AC, making it more sensitive for detecting presumed inflammatory cells in the aqueous humor, as hyper-reflective foci (HRF). In this study, we describe automated quantification of AC cells using SS AS-OCT in eyes with uveitis; validate the automated algorithm against manual counting of cells on SS AS-OCT images; and compare automated counts to SUN grades assigned clinically to the same eyes.

## Methods

Patients with histories of uveitis involving the anterior segment in one or both eyes were recruited from the practices of two uveitis specialists (GNH, ET) at the UCLA Jules Stein Eye Institute, University of California, Los Angeles. Patients were included as study participants whether or not uveitis was clinically active at the time of the study. Active uveitis was defined as a clinical AC cell grade of 0.5 + or higher, based on SUN criteria. If patients had unilateral disease, only the eye with uveitis was included in the study. Excluded were eyes that had ever undergone intraocular surgery. Also recruited were individuals who had normal eye examinations and no histories of intraocular inflammation, intraocular surgery, or ocular trauma; their eyes served as controls. The study was approved by the UCLA Health Institutional Review Board. Written informed consent was obtained from all study participants, unless they were < 18 years of age, in which case written informed consent was obtained from a parent of the study participant and an additional assent document was signed by study participants 7 years of age and older. The study adhered to the tenets of the Declaration of Helsinki.

### Data acquisition

AC cells were scored clinically for all study eyes according to SUN criteria [3] by one of the two aforementioned uveitis specialists during a comprehensive eye examination performed on the same day as SS AC-OCT imaging. Clinical grading was performed prior to image acquisition. Images were obtained by experienced operators using the ANTERION SS AS-OCT device (Heidelberg Engineering, Heidelberg, Germany). Both clinical grading and imaging were performed prior to pupillary dilation. A single 14 mm horizontal cross-sectional line B-scan was obtained just below the corneal apex, to avoid the apical light reflex; as such, no averaging was performed for this study. If scans were unclear because of poor focus or artifact from motion or corneal reflex, repeat imaging was attempted.

### Data analysis and statistical techniques

SS AS-OCT B-scan images were deidentified and exported in PNG format. An automated algorithm was developed internally by our research group for segmentation of AC structures and HRF detection in line-scan images using ImageJ version 1.54f (National Institutes of Health, Bethesda, MD; https://imagej.nih.gov/ij/index.html) and its plug-in software. Image processing was performed with binarization and morphology transformation to reduce noise interference. The Triangle thresholding algorithm was applied to generate a binarized image. To reduce speckle noise, a Gaussian blur smoothing operation was performed before the binarization. The AC space was segmented by automated contour detection, to include the entire area between the posterior corneal face and the anterior iris face and anterior lens capsule behind the pupillary space; the entire area was considered to be the region of interest (ROI). The algorithm used in this study identified and quantified hyperreflective signals that exceeded the mean gray value of the space outside of the cornea, which should include only speckle noise. A particle counter was used to tally the number of these signals for each scan and output the number as a final HRF count. Once the SS AS-OCT B-scan was exported, the implementation of the algorithm was fully automated; it did not require human intervention for selection of the ROI.

The same set of images were provided in random order to one investigator (SSG) for manual counting of the HRF on each image. The investigator was masked to the clinician-assigned SUN grade and to the automated HRF count for each image. By design, if there were more than 10 images for any SUN grade, only 10 random images for that grade were counted manually. The investigator used the same definition of an AC cell that has been reported in previous AS-OCT studies: hyperreflective dots in the AC that are brighter than the background noise or greater than two pixels in size [8]. Absolute agreement between the automated HRF counts and manual counts across all SUN grades was evaluated using intraclass correlation (ICC) derived from a two-way mixed-effects model using the HRF count method and SUN grade as fixed effects and patient random intercept. Pearson correlation coefficient was used to assess the consistency of agreement.

Mixed effects linear regression models were used to compare automated HRF counts to clinician-assigned SUN grades, with automated HRF counts and the log transformed automated HRF counts as the dependent variables, and the clinician-assigned SUN grades, age, sex, and laterality as the fixed effect; also included was a patient random intercept due to individual variability between eyes. A similar mixed-effects model was used to compare automated HRF counts for eyes with histories of uveitis, but for which no AC cells were seen on slit lamp biomicroscopy (Grade 0) and automated HRF counts performed on images from control eyes. All statistical tests were performed using R Statistical Software (v4.2.3; R Core Team 2023) with a two-tailed statistical significance set at *p* < 0.05 and 95% confidence interval.

## Results

**Fig. 1:**
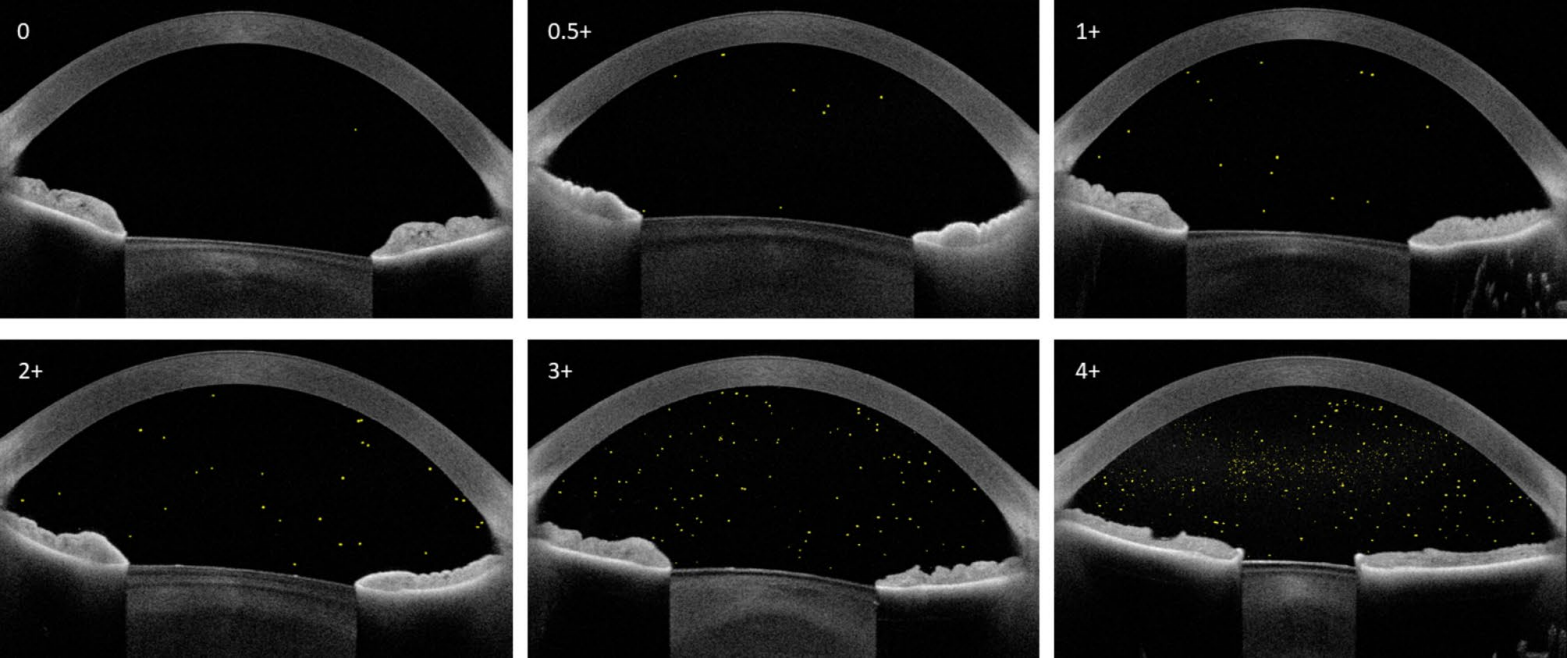
Images of swept-source anterior segment optical coherence tomography single line-scans obtained just below the apex of the cornea, to avoid the apical light reflex. A representative scan is shown for eyes in each Standardized Uveitis Nomenclature (SUN) grade, as assigned by one of two uveitis experts using slit lamp biomicroscopy. Yellow spots in the anterior chambers represent hyperreflective foci counted automatically by a software algorithm

Included were 90 eyes of 54 study participants; 67 eyes of 41 participants had histories of uveitis involving the anterior segment and 23 eyes of 13 participants with no history of eye diseases served as controls. Table 1 shows demographic and ophthalmic data for those patients with histories of uveitis. The mean age of control participants (25.2 ± 22.1 years) did not differ significantly from participants with uveitis (*p* = 0.88). The proportion of control participants who were female (7 participants, 54%) was smaller than for participants with uveitis, but the difference was not significant (*p* = 0.30). None of the control eyes had AC cells by slit lamp biomicroscopy. No eye with uveitis was noted to have an hypopyon on slit lamp biomicroscopy. Figure 1 shows representative line-scan images obtained with SS AS-OCT, corresponding to each clinician-assigned SUN grade of AC cells.

**Table 1:** Demographic and ophthalmic factors of 67 eyes (41 study participants) with histories of uveitis involving the anterior segments that underwent swept-source anterior segment optical coherence tomographic imaging

Factor	Individuals (*n* = 41)	Eyes (*n* = 67)
Age (years)		
Mean ± SD	26.1 ± 17.8	
Median (range)	17 (5–80)	
Sex (n [%])		
Female	30 (73)	
Male	11 (27)	
Diagnosis of uveitis (n [%])		
Idiopathic anterior uveitis	19 (46.3)	33 (49.3)
JIA-associated uveitis	8 (29.5)	11 (16.4)
Idiopathic panuveitis	5 (12.2)	9 (13.4)
HLA-B27-associated acute anterior uveitis	5 (12.2)	7 (10.4)
Blau syndrome	2 (4.9)	4 (6.0)
Idiopathic anterior and intermediate uveitis	1 (2.4)	2 (3.0)
HZO-associated anterior uveitis	1 (2.4)	1 (1.5)
SUN grade* (n [%])		
0 (no cells seen)		42 (62.7)
0.5+		10 (14.9)
1+		9 (13.4)
2+		3 (4.5)
3+		2 (3.0)
4+		1 (1.5)

HLA = human leukocyte antigen; HZO = herpes zoster ophthalmicus; JIA = juvenile idiopathic uveitis; SD = standard deviation; SUN = standardized uveitis nomenclature

* Determined clinically by one of two uveitis specialists using slit lamp biomicroscopy prior to imaging of the anterior chambers

**Fig. 2:**
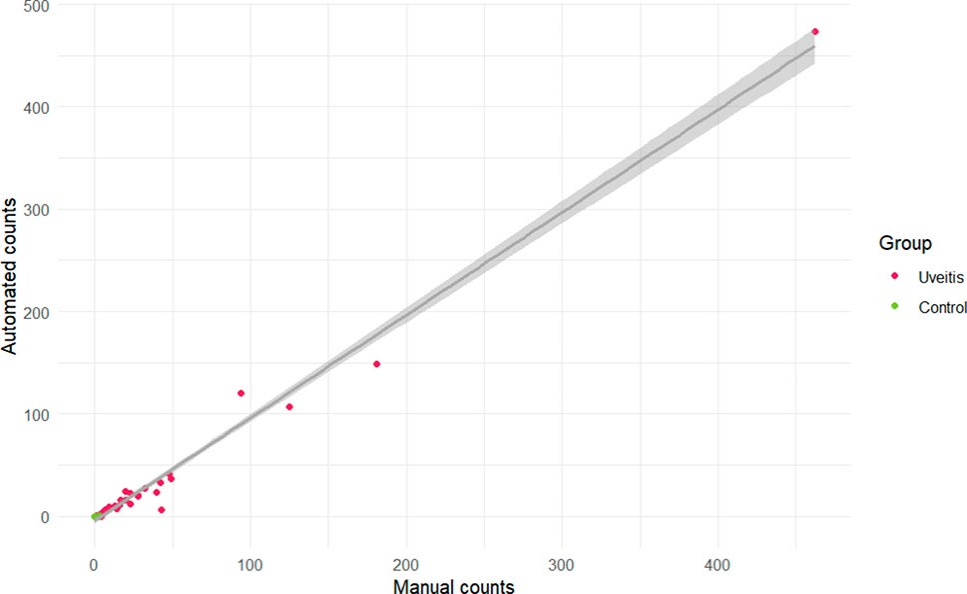
Scatter plot comparing manual versus automated counts of hyperreflective foci on the same set of single line-scan images obtained by swept-source anterior chamber OCT. The investigator who counted HRF manually was masked to SUN grades and automated counts. Interclass correlation is 0.99. The grey region in the plot represents the confidence interval (CI) for the estimated regression line

Table 2 shows manual and automated counts for eyes with uveitis, grouped by clinician-assigned SUN grades. There was a strong correlation between manual and automated counts across all grades (ICC = 0.99, *r* = 0.98), as shown in Fig. 2.

**Table 2:** Comparison of manual counts to automated counts of anterior chamber hyper-reflective foci using the same set of single-line scans generated by swept-source anterior segment optical coherence tomography performed on 67 eyes (41 study participants) with uveitis involving the anterior segment grouped by clinician-determined Standardization of Uveitis Nomenclature clinical grades

SUN Clinical Grade	Hyper-reflective Foci*						
	Manual Counts^†§^				Automated Counts^‡§^		
	Scans Counted	Median	IQR	Range	Median	IQR	Range
0^¶^	10	3	2,4	0–6	2	0,4	0–11
0.5+	10	15	10,17	7–28	10	8,15	6–21
1+	9	41	27,44	23–49	22	15,33	6–41
2+	3	32	--	20–94	27	--	24–120
3+	2	153	--	125–181	128	--	107–149
4+	1	462	--	--	474	--	--

IQR = interquartile range; SUN = standardized uveitis nomenclature

* Hyper-reflective foci were detected with ANTERION swept-source anterior segment optical coherence tomography device (Heidelberg Engineering, Heidelberg, Germany). Images showed the entire depth and width of the anterior chamber

† Manual counts were performed by a single masked investigator using the same set of line-scans counted automatically with the study algorithm. All scans were counted for SUN clinical grades 0.5 + and higher; a random selection of 10 scans were counted for grade 0

‡ Fully automated counts used a software algorithm on a single line-scan image obtained just below the corneal apex to avoid the apical light reflex for each study eye

§ Interclass correlation for comparison of manual to automated counts across all grades was 0.99

¶ The SUN Working Group defines Grade 0 as < 1 cell per 1 mm x 1 mm field; no cells were seen clinically in these 42 eyes and uveitis was considered to be clinically inactive

**Fig. 3:**
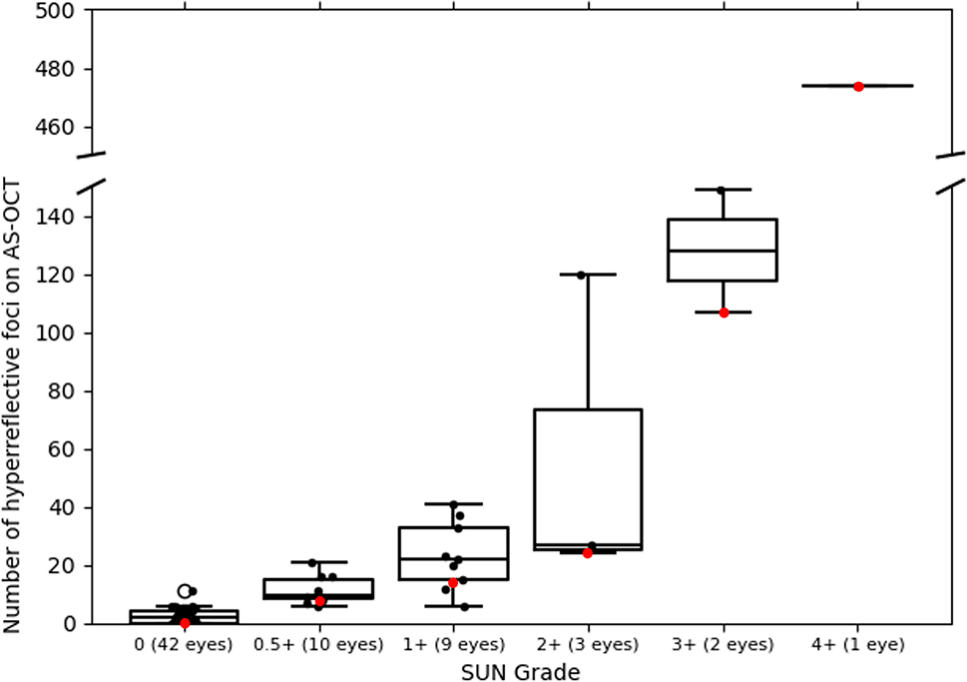
Box-and-whisker plot showing automated counts of hyperreflective foci on single line-scan images obtained by swept-source anterior chamber OCT grouped by clinician-assigned Standardized Uveitis Nomenclature (SUN) grades. Lines inside the boxes represent medians. Boxes identify the first and third quartiles. Red dots in the box-and-whisker plot reflect the representative examples seen in Fig. 1. There was a significant correlation between automated counts and SUN grades (Pearson correlation coefficient = 0.80, *p* < 0.001)

There was a strong positive correlation between automated counts and clinician-assigned SUN grades (Pearson correlation coefficient = 0.80, *p* < 0.001), as shown in Fig. 3. When adjusted for age, sex, and laterality, the number of HRF increased by 38 units (*p* < 0.001) or 293% (*p* < 0.001) for each 1-step increase in SUN grade. Some eyes for which no cells were seen by slit lamp biomicroscopy (SUN grade 0) were determined to have HRF by SS AC-OCT; the automated counts for these eyes (median 2 HRF [IQR 0,4]) were significantly higher than the automated counts for control eyes (median 0 HRF [IQR 0,1], *p* = 0.02).

## Discussion

The fully automated HRF counts produced by our algorithm agreed strongly with manual counts using the same set of high-resolution SS AS-OCT line-scan images of anterior chambers from eyes with histories of uveitis, providing a measure of technical validity for our method. There was a significant correlation between the automated HRF counts and clinician-assigned SUN grades, after adjusting for age, sex, and laterality, but there was substantial within-grade variation in the automated HRF counts. Unlike SUN grades, which are ordinal and subjective, AS-OCT-based quantification provides a continuous and objective measure, allowing greater precision of counts. The ability to distinguish small changes in cell number is likely to be particularly important during longitudinal assessments of chronic uveitis, which tends to have levels of inflammation corresponding to the lower SUN grades. Validation against definitive clinical outcomes will be needed to confirm the value of such measurements.

Various approaches have been used to perform objective quantification of AC cells using AS-OCT. First generation AS-OCT devices employed a time-domain (TD) system. One such device is the Visante (Carl Zeiss Meditec AG, Jena, Germany), which operated at a central wavelength of 1310 nm, but with low axial resolution (18–25 μm) and a low scan speed (2000 A-scans/second). Agarwal and associates first used TD AS-OCT to quantify AC cells in 2009 [9]. Li and associates also used TD OCT in a study that showed correlations of imaged cells with in vitro measurements and with clinician-associated grades [10]. The TD AS-OCT technique is limited, however, by its axial and transverse resolutions, which are larger than individual white blood cells (which can be as small as 7–9 μm). The advent of the spectral-domain (SD) AS-OCT addressed the limitations of TD technology by using a higher image acquisition speed and a better axial resolution. Using SD-OCT-based quantification of AC cells, Sharma and associates found a strong correlation between imaging results and clinician-assigned SUN grades [11]. Nevertheless, SD AS-OCT devices, utilizing a shorter wavelength light source (around 840 nm), have constraints in image depth range and have loss of signal strength with depth.

Our approach has the following unique advantages: (1) it utilizes high-resolution SS AS-OCT images, which provide visualization of the entire AC; and (2) the fully automated algorithm is able to delineate all anterior segment structures, select the ROI, and quantify HRF without the need for manual intervention. The integration of a longer wavelength light source with SS AS-OCT has proved transformative, allowing for greater image depth and high-contrast imaging of the entire anterior segment, spanning from the cornea to the posterior surface of the lens. The ANTERION device has a 1300 nm light source and a speed of 50,000 A-scans/second, which allows for the acquisition of high-contrast AS images with an image depth of 14 mm, a lateral width up to16.5 mm, an in-tissue axial resolution of < 10 μm and lateral resolution of 30–45 μm. There is minimal sensitivity roll-off along a large dynamic range, resulting in high-contrast images of the entire AS and the posterior lens [12]. The result is detailed visualization and analytics of AS structures, presumably providing an advantage in the precision of AC cell quantification, compared with earlier generation AS-OCT devices with SD imaging.

Many previous studies of imaging-based assessment of AC cells employed manual quantification of HRF [8, 13–15]. Several semi-automated methods have also been published [16–21], generally necessitating the manual segmentation of the ROI, usually representing only part of the AC imaged in the scan. Basing counts on a non-standardized, clinician-assigned ROI that includes only a partial area of the AC introduces the possibility of user bias, especially considering the fact that inflammatory cells may not distribute evenly in the AC [22]. An additional disadvantage is the time needed for a skilled individual to select the ROI manually. Fully automated algorithms have been used in a few previous studies of AC cell quantification, but those studies utilized TD [10] or SD [11, 23] AS-OCT devices. Keino and associates quantified presumed AC cells in 48 eyes with uveitis using the CASIA 2 SS AS-OCT and a fully automated algorithm, but did not include a comparison to control eyes [24]. 

A potential advantage of AS-OCT-based imaging of AC cells is the potential to detect subclinical disease, as suggested in previous studies [10, 11, 16, 22]. Likewise, in our study, some of the eyes with histories of uveitis for which no cells were seen on clinical examination had HRF detection on SS AS-OCT images. Solebo and colleagues used the ANTERION device to obtain AC scans on 434 eyes of 217 children without uveitis. Images were manually graded by at least two independent readers for HRF, and when discrepancies between graders occurred (1.8% of total scans), those images underwent semi-automated analysis [22]. The number of HRF ranged from 0 to 6, with at least one HRF in 76% of scans. The authors postulated that the HRF might represent pigment or other non-inflammatory particles; slit lamp biomicroscopy was not performed in their study. In our study, the fact that there was a small, but significant difference in HRF counts between control eyes and those with histories of uveitis but no clinically detected cells suggests that at least some of the HRF represent leukocytes and that SS AS-OCT quantification will be more sensitive than clinical examinations for detecting minimal, residual inflammation in treated eyes or subclinical disease on initial examination of a patient suspected of having uveitis. Conversely, because a few control eyes had up to 5 HRF, it is possible that these low levels of HRF may represent a physiologic, rather than a disease, state in some individuals. Longitudinal studies will be needed to determine the relevance of subclinical HRF.

Our study is limited by its relatively small sample size, especially at HRF counts corresponding to the highest SUN grades; however, precision at the high cell levels, which may be encountered at initial evaluations and the start of treatment, is less important than precision at lower levels of inflammation, when small changes in cell count may have important clinical implications. Our inability to discriminate between leukocytes and other particles, such as pigment, when arriving at HRF counts is another limitation of AS-OCT imaging techniques. Our study suggests future investigations that can address these and other issues must be considered before AS-OCT assessment of anterior segment inflammation can be implemented into clinical trials and routine clinical practice. Repeated imaging from eyes will assess reproducibility of measurements. Hyperreflective foci can appear as different sizes which likely reflect different types of white blood cells and future studies will aim to identify the subtype of white blood cell based on hyperreflective foci size and brightness profile. Study of other HRF characteristics, such as size and density, may help to identify leukocytes from other particulate matter in the AC. With regard to the unequal distribution of cells in the AC of inflamed eyes [22], it will need to be determined whether scans across the central AC, as in our study, are more predictive of outcomes than would be scans from other areas. Longitudinal evaluations will also determine whether there are HRF count thresholds that best predict development of ocular complications or vision loss. With regard to the possible identification of subclinical disease, studies should evaluate the opposite eyes of patients thought clinically to have unilateral uveitis.

In summary, we have demonstrated that our fully automated algorithm can reliably count HRF in the AC when compared to manual counts on line-scan images obtained with high-resolution SS AS-OCT, and that automated counts correlate with clinician-assigned SUN grades. Images are acquired quickly in a noncontact fashion, with lower illumination than generally used during slit lamp biomicroscopy, and in contrast to clinical assessments, counts are precise and linear. With additional study, automated counting of AC cells using SS AS-OCT will likely provide accurate, reproducible, and standardized assessments that will improve evaluation and treatment of uveitis. Among the potential benefits is an ability to identify sub-clinical levels of anterior segment inflammation.

## Electronic supplementary material

Below is the link to the electronic supplementary material.

Supplementary Material 1

## Data Availability

No datasets were generated or analysed during the current study.
