# Supplementary figures and images for: Automated quantification of anterior chamber cells using swept-source anterior segment optical coherence tomography
Source: J Ophthalmic Inflamm Infect. 2025 Jan 9;15:3. doi: 10.1186/s12348-025-00456-y (PMC11717729; doi:10.1186/s12348-025-00456-y)

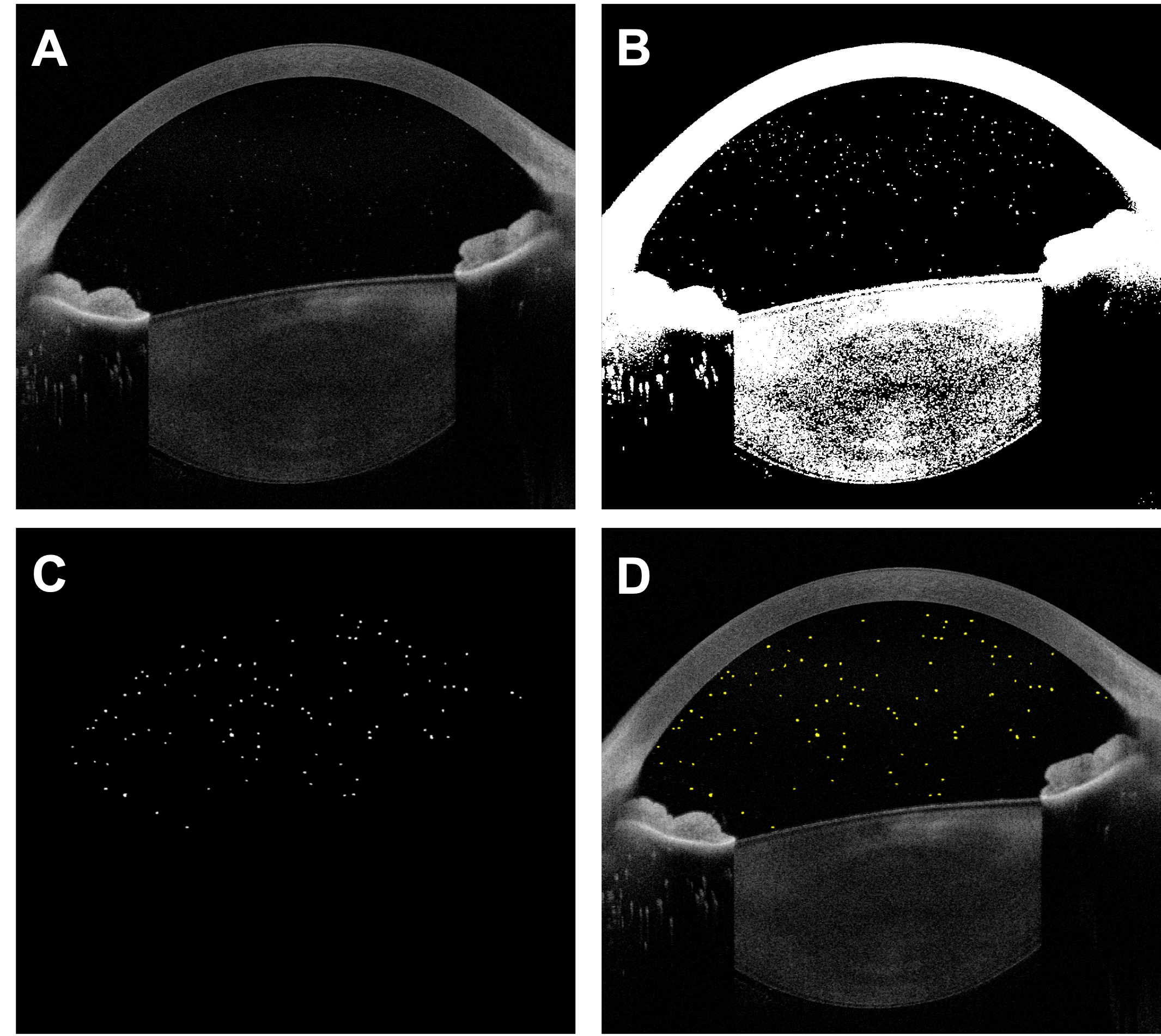

Supplement: Supplementary file 1 — Supplementary Material 1 [file 12348_2025_456_MOESM1_ESM.tiff]
